# The Frequency of Antibiotic Resistance and ESBLs Among Clinically *Acinetobacter baumannii* Strains Isolated from Patients in a Major Hospital in Tehran, Iran

**DOI:** 10.2174/1874285801812010254

**Published:** 2018-07-31

**Authors:** Reza Ranjbar, Sajjad S. Tolon, Shahin Zayeri, Mehrdad Sami

**Affiliations:** 1 Molecular Biology Research Center, Systems Biology and Poisonings Institute, Baqiyatallah University of Medical Sciences, Tehran, Iran; 2 Department of Clinical Sciences, School of Veterinary Medicine, Ferdowsi University of Mashhad, Mashhad, Iran

**Keywords:** Antibiotic, Bacterial resistance, *A. baumannii*, ESBL, PCR, Clinical samples

## Abstract

**Background:**

Bacterial resistance to antibiotics limits treatment options, increases morbidity and mortality, and raises the risk of antibiotic-associated adverse events. Antibacterial resistance emerges rapidly following an increase in the consumption of antibiotics against infectious diseases. The spread of ESBL producing strains has a limiting factor based on antibiotic function for the treatment of infections particularly caused by *Acinetobacter baumannii* (*A. baumannii*).

**Objective:**

This study was conducted to evaluate the prevalence of antimicrobial resistance and distribution of *bla_TEM_*, *bla_CTX_*, and *bla_SHV_* genes among *A. baumannii* strains isolated from clinical samples at a major hospital in Teheran, Iran.

**Methods:**

*A. baumannii* strains were isolated and identified using standard microbiological methods. The disc diffusion and combined discs methods were used for testing antimicrobial susceptibility and to identify the strains producing Extended-Spectrum Beta-Lactamases (ESBL), respectively. DNA extraction was done by boiling method. Finally, the frequency of resistant genes including *bla_TEM_*, *bla_CTX_*, and *bla_SHV_* in ESBL producing isolates was studied by PCR.

**Results:**

Gender distribution in this study was 53 (53%) samples for men and 47 (47%) for women. Totally, one hundred *A. baumannii* strains were isolated. More than 93% of the isolates were multi drug resistant. The highest to lowest antibiotic resistance was observed against amoxicillin/clavulanic acid (98%), ceftriaxone (96%), cefotaxime (94%), and ceftazidime (93%), respectively. The frequency of positive phenotypic test of ESBL was 19% and 16% for CAZ-C and CTX-C, respectively. The frequency of *bla_TEM_*, *bla_CTX_*, and *bla_SHV_* genes was 52.1, 43.4, and 21.7, respectively.

**Conclusion:**

*A. baumannii* isolates exhibited an extremely worrying level of antibiotic resistance, and a high percentage of the isolates showed MDR in this study. This is a serious warning because ESBLs are a major threat to the effectiveness of antibiotics that are currently available for medical uses. The frequency of genes encoded ESBL isolates of *A. baumannii* may be due to overuse and misuse of antibiotics.

## 1 INTRODUCTION

Bacterial resistance to antibiotics limits treatment options increases mortality and the risk of side effects of antibiotics. Resistance to excessive antibiotic use is increasing rapidly [1]. Extended-spectrum beta-lactamases (ESBL) is responsible for creating resistant strains of bacteria against antibiotics [2-4]. Expansion of ESBL producing strains restricted the antimicrobial agents to treat patients effectively and raised concerns for control of infections caused by *A. baumannii*. The misuse of antimicrobial drugs in hospitals can lead to the development of antibiotic-resistant bacteria such as *A. baumannii*. Acinetobacter's hospital strains are usually resistant to several drugs at the same time. *A. baumannii* is more common than other Acinetobacter strains and is the most common cause of bacterial infection. This opportunistic bacteria can survive on a variety of surfaces, including patient skin and medical equipment. Therefore, prolonged hospitalization in the intensive care unit, severe illnesses, immunodeficiency conditions, burn injuries, long-term use of antimicrobial agents, and catheter use are some of the most important risk factors for Acinetobacter infection [5]. *A. baumannii* is an important opportunistic pathogen responsible for a variety of nosocomial infections; including ventilator-associated pneumonia, bacteremia, surgical site infections, secondary infections of burn patients, secondary meningitis, and urinary tract infections in the Middle East region [6, 7] and other parts of the world [8, 9]. Hydrolysis of the β-lactam ring structure by ESBL causes an abnormal antibiotic function. ESBL enzymes are able to target a wide range of antibiotics, such as penicillins and their complexes, monobactams, and new generation cephalosporins [10]. There are hundred types of β-lactamase enzymes, each of the ESBL bacteria may have genes for one or more of these enzymes. Similar *bla* genes, similar to the same structure within these gene families, are grouped according to phylogenetic and are often detected and diagnosed by PCR [11]. Transmission of resistance genes between bacteria is easy to do since these genes are encoded on mobile vectors (plasmids and transposons) [12, 13]. β-lactamases are inactivating enzymes that are present in all types of bacteria. The efficacy of beta-lactam antibiotics in eradicating infection in different places in the body can be reduced by the beta-lactamase producing organisms [14]. By using DNA-based technologies, a new era in the diagnostic and molecular epidemiology of an antibiotic-resistant bacterium opens. The use of PCR-based techniques has revolutionized the rapid diagnosis of determinants of resistance, such as ESBLs [15, 16].

We have previously investigated ESBLs genes in some high resistant bacterial species particularly those were isolated from clinical cases [17-22].

In this study, we aimed to determine the prevalence of antimicrobial resistance and distribution of *bla_TEM_*, *bla_CTX_*, and *bla_SHV_* genes among *A. baumannii* strains isolated from clinical samples at a major hospital in Teheran, Iran.

## 2 MATERIALS AND METHODS

In a cross-sectional descriptive study, from January until the end of June 2017, clinical samples (endotracheal tubes, blood, sputum, urine, catheters, wounds, and various fluids) were recovered from patients admitted to a major Hospital in Tehran.

### 2.1 Bacterial Strains Identification

The samples were transported to the laboratory then were cultured on blood and MacConkey agar for 24 hours at 37°C was done. To identify the isolates of *A. baumannii,* the preliminary conventional phenotypic tests including growth on MacConkey agar, sugar fermentation, motility, catalase and oxidase tests, and other standard recommended tests [23, 24]. For definitive identification of these isolates to the species level, molecular methods were used in the next step.

### 2.2 Antibiotics Susceptibility Testing

According to the instructions of the clinical and laboratory standards institute, antibiotic resistance of the isolates was determined using the disk diffusion antibiotic sensitivity test [25]. Antibiotic discs used in this study were including cefotaxime, ceftazidime, amoxicillin/clavulanic acid and ceftriaxone. *A. baumannii* suspension was expanded after incubation at 37°C on Mueller-Hinton agar culture medium and compared to McFarland standard using carpet culture. Then the discs were placed on them. After incubation for 24 hours, the growth inhibition diameter was measured and compared with reference values.

### 2.3 Detection of ESBL Producing Strains

The phenotypic detection of the different beta-lactamases was performed on Mueller-Hinton medium using discs containing ceftazidime, ceftazidime/clavulanic acid (CAZ-C), cefotaxime, and cefotaxime/clavulanic acid (CTX-C) as previously described [26].

The DNA was extracted from isolates by the boiling method and frequency of *TEM*, *SHV* and *CTX* using specific primers was evaluated, as shown in Table **1** [26, 27]. PCR test was performed using Master Mix, primer, bacterial DNA, and distilled water to obtain a final volume of 25 mL. Except for annealing temperature, the reaction temperature of the PCR for all three beta-lactamase genes was the same. It contained an initial denaturating for 4 minutes at 94°C, followed by 35 repetitions for 1 minute at 94°C, amplification at 72°C for 40 seconds, and final amplification at 72°C for 5 minutes (Table **1**). PCR products were electrophoresed on 1.5% agarose gel containing ethidium bromide at 80 V for 1 h.

## 3 RESULTS

**Fig. (1):**
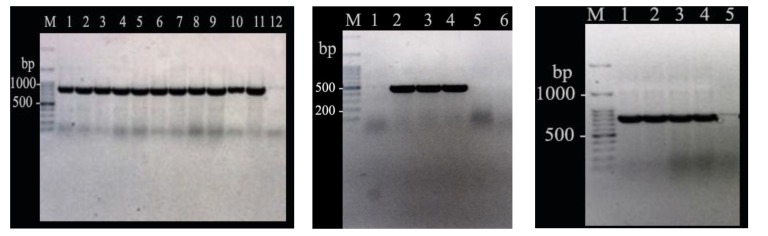
PCR amplification products of *bla_TEM_*, *bla_CTX_,* and *bla_SHV_* (In order from left to right) in some representative *Acinetobacter baumannii* strains.

Totally, one hundred *A. baumannii* strains were isolated. Gender distribution in this study was 53 (53%) samples from men and 47 (47%) of women. The percentage of antibiotic resistance was: amoxicillin/ clavulanic acid (98%), ceftriaxone (96%), cefotaxime (94%), and ceftazidime (93%). Of these, 93% of the clinical samples showed multiple antibiotic resistance. The frequency of positive phenotypic test of ESBL was 19% and 16% for CAZ-C and CTX-C, respectively. The frequency of resistance genes was 52.1, 43.4, and 21.7 in *A. baumannii* isolates based on the detection of ESBLs genes including *bla_TEM_*, *bla_CTX_,* and *bla_SHV_*, respectively (Fig. **1**).

## 4 DISCUSSION

In this study, antimicrobial susceptibility testing of *A. baumannii* isolates originally showed highly significant resistance to different types of antibiotics. This resistance can be due to the presence of specific genes of ESBL such as *bla_TEM_*, *bla_CTX_*, and *bla_SHV_*. Knowing the types and frequency of these genes helps us to make a good decision for the treatment process of patients effectively. High level of multi-drug resistance including Cefotaxime (CTX), Ceftazidime (CAZ), Amoxicillin/clavulanic acid (AMC) and Ceftriaxone (CRO) was observed among the isolates under the study.

Various factors, including the abuse of antibiotics, the spread of clonal resistant microorganisms, can cause the release of highly resistant pathogens to the environment and clinical setting. Previous researches showed that the prevalence of *A. baumannii* MDR isolates ranged from 32.7% to 100% [28-32].

Among the mechanisms that create resistance to drugs, ESBLs play an important role in resistance to conventional antibiotics such as penicillin and cephalosporins. ESBL genes, due to the widespread diffusion of pathogens from the hospital through plasmids and integrons, can further increase drug resistance, including MDR isolates [34, 35].

Jaggi *et al*. reported that the prevalence of some cephalosporins antibiotics group resistance in the *A. baumannii* strains of clinical samples against cefepime, ceftazidime, and ciprofloxacin was 90.3%, 92.1%, 91.6%, respectively [36]. Similar findings have been reported from different countries [37-40].

Several studies have reported the frequency of ESBL gene in *A. baumannii* ranging from 25% to 93.5% [41-44]. In the present study, of the 100 isolates of *A. baumannii*, 39% of the ESBL producer was consistent with the results of previous studies. Safari *et al*. reported that *SHV* (58%) and *TEM* (20%) were the highest numbers of ESBL genes in their study [34]. Azhar *et al*. based on a study that conducted in Iraq, reported that *SHV* (25%) was the most frequently detected ESBL gene [45], while in this study, *TEM* (52.1) was the most. Khalilzadegan and colleagues identified that *CTX*-M and *TEM* have most ESBL genes [31]. There is some reason to observe the differences in resistance patterns and the prevalence of *A. baumannii* in various investigations including abuse and misuse of antibiotics, differences in the type of antibiotics used, long-term hospitalization, type of samples taken, differences in diagnostic methods used to identify genes, geographical conditions, gender and age of patients, and so on [46, 47].

This study showed high levels of antibiotic resistance in *A. baumannii* isolates recovered from clinical samples. This is a serious warning because ESBLs are a major threat to the effectiveness of antibiotics that are currently available for medical uses. In this paper, we presented evidence of a great frequency of multi-drug resistant bacteria in clinical samples. Resistance to antimicrobials increasingly undermines the effectiveness of the use of b-lactam antibiotics and other antibacterial drugs that have been effective in the past. To combat antibiotic resistance and develop knowledge-based interventions, a comprehensive understanding of the mechanisms of resistance and the regulation of genes that cause resistance to the material is needed. Molecular methods particularly DNA-based detection techniques help us in the field of diagnostic and molecular epidemiology of antibiotic resistance genes [48-50].

## CONCLUSION

*A. baumannii* isolates exhibited an extremely worrying level of antibiotic resistance, and a high percentage of the isolates showed MDR in this study. This is a serious warning because ESBLs are a major threat to the effectiveness of antibiotics that are currently available for medical uses. The frequency of genes encoded ESBL isolates of *A. baumannii* may be due to overuse and misuse of antibiotics.

## Figures and Tables

**Table 1:** Primers used to amplify the genes of beta-lactamase.

Target type of β-lactamase Genes	Primer Sequence	Size of Product (bp)	AT (*ºC*)
*bla_TEM_*	5´-ATG AGT ATT CAA CAT TTC CG-3´ (F)	867	52.2
	5´-CTG ACA GTT ACC AAT GCT TA-3´ (R)		
*bla_CTX_*	5´-TTT GCG ATG TGC AGT ACC AGT AA- 3´ (F)	560	60
	5´-CTC CGC TGC CGG TTT TAT -3´ (R)		
*bla_SHV_*	5´-TTA ACT CCC TGT TAG CCA-3´ (F)	860	51.2
	5´-GAT TTG CTG ATT TCG CCC-3´ (R)		

F: Forward; R: Reverse; AT: Annealing temperature.
